# Nuc2p, a Subunit of the Anaphase-Promoting Complex, Inhibits Septation Initiation Network Following Cytokinesis in Fission Yeast

**DOI:** 10.1371/journal.pgen.0040017

**Published:** 2008-01-25

**Authors:** Ting Gang Chew, Mohan K Balasubramanian

**Affiliations:** Cell Division Laboratory, Temasek Life Sciences Laboratory and the Department of Biological Sciences, National University of Singapore, Singapore; National Institute of Diabetes & Digestive & Kidney Diseases, United States of America

## Abstract

In most cell types, mitosis and cytokinesis are tightly coupled such that cytokinesis occurs only once per cell cycle. The fission yeast *Schizosaccharomyces pombe* divides using an actomyosin-based contractile ring and is an attractive model for the study of the links between mitosis and cytokinesis. In fission yeast, the anaphase-promoting complex/cyclosome (APC/C) and the septation initiation network (SIN), a spindle pole body (SPB)–associated GTPase-driven signaling cascade, function sequentially to ensure proper coordination of mitosis and cytokinesis. Here, we find a novel interplay between the tetratricopeptide repeat (TPR) domain–containing subunit of the APC/C, Nuc2p, and the SIN, that appears to not involve other subunits of the APC/C. Overproduction of Nuc2p led to an increase in the presence of multinucleated cells, which correlated with a defect in actomyosin ring maintenance and localization of the SIN component protein kinases Cdc7p and Sid1p to the SPBs, indicative of defective SIN signaling. Conversely, loss of Nuc2p function led to increased SIN signaling, characterized by the persistent localization of Cdc7p and Sid1p on SPBs and assembly of multiple actomyosin rings and division septa. Nuc2p appears to function independently of the checkpoint with FHA and ring finger (CHFR)–related protein Dma1p, a known inhibitor of the SIN in fission yeast. Genetic and biochemical analyses established that Nuc2p might influence the nucleotide state of Spg1p GTPase, a key regulator of the SIN. We propose that Nuc2p, by inhibiting the SIN after cell division, prevents further deleterious cytokinetic events, thereby contributing to genome stability.

## Author Summary

Cytokinesis is the process by which a mother cell is physically partitioned into two daughter cells. Cytokinesis is well coordinated with segregation of the genetic material to ensure that the genome is not damaged by the cell division apparatus. How untimely cytokinesis is prevented is not fully understood, and is a topic of current interest. Studies of the mechanisms of segregation of the genetic material and cytokinesis have benefited extensively from the use of the fission yeast *Schizosaccharomyces pombe.* In this study, we make the discovery that fission yeast Nuc2p, a protein previously known to form part of a multi-protein machine that regulates genome segregation, has a second function in regulating cytokinesis. Nuc2p appears to dampen the septation initiation network, which is an important signaling pathway that is essential for cytokinesis. Thus, Nuc2p prevents the occurrence of cytokinetic events prior to segregation of the genetic material and thereby contributes to genome stability. Since the multi-component machinery that Nuc2p forms part of, as well as Nuc2p itself, has relatives in essentially all eukaryotic cells, a similar mechanism might operate in other cells as well.

## Introduction

The eukaryotic cell cycle is composed of an invariant sequence of events, in which DNA replication precedes mitosis and mitosis in turn precedes cytokinesis [1]. Cells also possess mechanisms to ensure that DNA replication, chromosome segregation and cytokinesis occur only once per cell cycle [2,3]. While much progress has been achieved in understanding the temporal regulation of DNA synthesis and chromosome segregation, the mechanisms by which cytokinesis is restricted to once per cell cycle has not been fully explored.

In recent years, the fission yeast Schizosaccharomyces pombe has emerged as an attractive organism for the study of cytokinesis and its relation to the rest of the cell cycle [4]. S. pombe cells, like animal cells, divide utilizing an actomyosin based contractile ring [5–15]. The actomyosin ring is assembled upon entry into mitosis and prior to chromosome segregation and it contricts after chromosome segregation and mitotic spindle disassembly [16,17].

In fission yeast, a signaling pathway known as Septation Initiation Network (SIN) is a key determinant of cytokinesis [4,7,18]. While loss of SIN function leads to an inability of cells to undergo cytokinesis, ectopic activation of SIN allows cytokinesis to proceed even prior to entry into mitosis [3,7,19]. SIN is a GTPase-driven signaling cascade that comprises a small GTPase, Spg1p, three protein kinases: Cdc7p, Sid1p, Sid2p, and their associated factors: Cdc14p, Mob1p [20–25]. This pathway is negatively regulated by a two-component GTPase Activating Protein (GAP), Cdc16p and Byr4p [26–29]. Components of the SIN localize to the spindle pole body (SPB) by association with the scaffold proteins Cdc11p and Sid4p [30–33]. In addition, two of the SIN components, Sid2p and Mob1p, also localize to the cell division site during cytokinesis [24,25]. Although most components of the SIN are detected at the SPB throughout the cell cycle, Cdc7p and Sid1p are detected at the SPB only during mitosis and cytokinesis [18,22,34,35]. In particular, since the localization of Sid1p to the SPB depends on cyclin B proteolysis and cyclin dependent kinase (CDK) inactivation, it has been proposed that the SIN might link cytokinesis to mitotic exit [22]. Proteolysis of cyclin B (and thereby the inactivation of CDK activity) is triggered by a multisubunit E3 ubiquitin ligase, termed the anaphase-promoting complex/cyclosome (APC/C) [36,37]. After completion of cytokinesis, the SIN pathway is inactivated, as characterized by the presence of Cdc16p and Byr4p on the SPB and the concomitant loss of Cdc7p and Sid1p from the SPB [18,38,39]. How the SIN pathway is inactivated following cytokinesis and how its precocious activation in interphase is prevented, while the CDK activity is low, remain poorly understood.

In this study, we uncover a function of Nuc2p, a Tetratricopeptide repeat (TPR)-domain containing subunit of APC/C, in preventing inappropriate cytokinetic events following cell division. While loss of Nuc2p function leads to uncontrolled septation, the current study and a previous study have shown that overproduction of Nuc2p leads to inhibition of cytokinesis [40]. Nuc2p appears to exert its effects on cytokinesis by modulating the nucleotide state of the Spg1p-GTPase and thereby down regulating the SIN.

## Results

### *nuc2-*663 Cells Undergo Multiple Septation Events at the Restrictive Temperature

**Figure 1:**
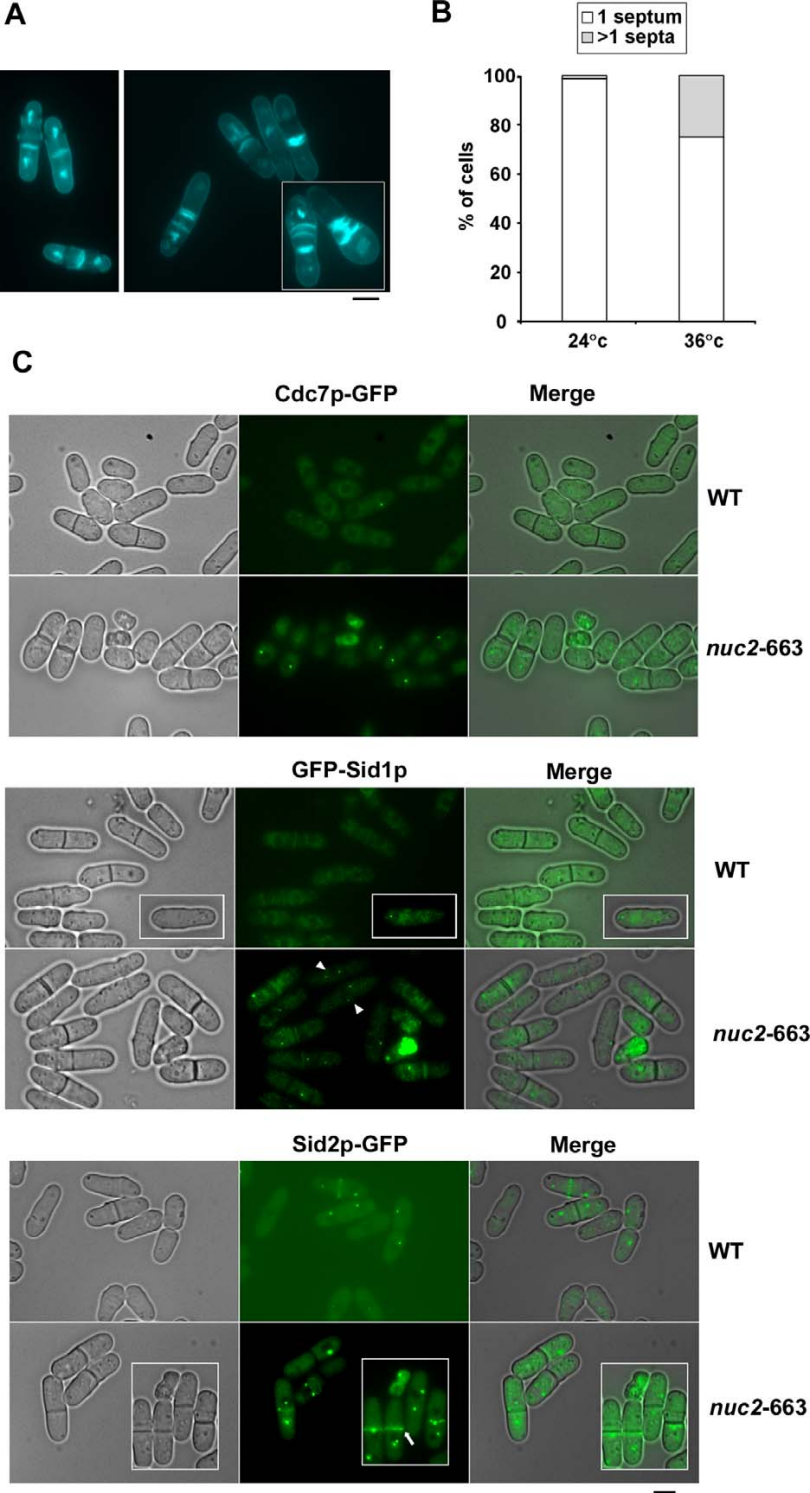
Hyperactivation of SIN Signaling in *nuc2-*663 Mutant (A) *nuc2-*663 cells were stained with DAPI and aniline blue to visualize nuclei and septum material, respectively. The left panel shows *nuc2*-663 cells at permissive temperature. The right panel shows *nuc2*-663 cells at restrictive temperature. (B) Quantification of multiseptated cells in the *nuc2-*663 mutant. (C) Wild-type and *nuc2-*663 cells expressing GFP tagged versions of Cdc7p, Sid1p, and Sid2p were grown at 36 °C for 4 h and were visualized using fluorescence microscopy. Arrowheads indicate mitotic cells with Sid1p at both SPBs. Arrows indicate cells in which Sid2p-GFP signal persisted at the division site after completion of cytokinesis. Scale bar, 5 μm.

Previous studies have shown that cells overproducing Nuc2p are not defective for mitotic exit, but die as elongated multinucleate cells [40]. Based on this observation it has been suggested that Nuc2p, a TPR-domain containing subunit of APC/C, is an inhibitor of septation [40]. To understand how Nuc2p functions in cytokinesis, we investigated the organization and function of the cytokinetic machinery in the *nuc2-*663 mutant. As previously reported, *nuc2-*663 cells, upon shift to the restrictive temperature, undergo cytokinesis in the absence of chromosome segregation [41]. Interestingly, we observed that approximately 28% of *nuc2-*663 cells (*n* = 112/404) displayed multiple septa, indicating that the cytokinetic machinery might be constitutively active in these cells (Figure 1A and 1B).

Since hyperactivation of SIN signaling also results in multiseptated cells, we tested the possibility that SIN signaling is up-regulated in the *nuc2-*663 mutant. Previous studies have shown that Cdc7p and Sid1p are localized to the SPBs in mitotic cells and are lost from the SPBs upon completion of cytokinesis in fully septated cells [18]. Accordingly, in wild-type cells, Cdc7p and Sid1p were detected at the SPBs in cells undergoing cytokinesis and were not detected at the SPBs in fully septated cells (Figure 1C, *wt* panel). Interestingly, in *nuc2-*663 cells shifted to the restrictive temperature, Cdc7p and Sid1p persisted at the SPBs even after completion of septation (Figure 1C, *nuc2-*663 panel). In addition, Sid1p was routinely detected on both SPBs in *nuc2-*663 cells shifted to the restrictive temperature (Figure 1C, indicated by arrowhead). This effect of persistent localization of Cdc7p and Sid1p in septated cells was similar to that observed in cells defective for Cdc16p function [22,35]. The protein kinase Sid2p and its binding partner Mob1p appear to constitute the most downstream elements of the SIN. In wild type cells, Sid2p and Mob1p localize to the SPBs throughout the cell cycle and are also detected at the cell division site during cytokinesis [24,25]. Interestingly, in the *nuc2-*663 mutant, Sid2p was detected at the division site in fully septated cells (Figure 1C, *nuc2-*663 panel; indicated by arrow). Furthermore, additional ring like structures containing Sid2p were also detected in these cells (Figure 1C). Similar localization of Sid2p was not seen in wild type cells, in which the medial localization of Sid2p was lost upon completion of septation (Figure 1C, *wt* panel). The persistent localization of Sid1p and Cdc7p to the SPBs and Sid2p at the division site in *nuc2-*663 cells suggested that Nuc2p might be involved in down regulation of SIN function following cytokinesis.

### Nuc2p Prevents Inappropriate Cytokinesis After Septum Assembly

The *nuc2-*663 mutant has been shown to be capable of partially proteolyzing the mitotic B-type cyclin, Cdc13p [42]. Since cyclin proteolysis as well as CDK inactivation is sufficient for completion of cytokinesis, it was possible that the multiseptated phenotype we observed was purely due to partial proteolysis of cyclin B and/or re-entry into a subsequent round of mitosis. To address if Nuc2p played a role in the regulation of cytokinesis after execution of its function in mitotic exit, we inactivated Nuc2p function after passage through anaphase. To this end, we generated a *nuc2*-663 *nda3*-KM311 strain so as to allow for the inactivation of Nuc2p function following anaphase. The *nda3* ^+^ gene encodes the β-subunit of the tubulin heterodimer and the *nda3*-KM311 mutant results in cold-sensitivity and lethality [43]. The *nda3*-KM311 allows the synchronization of cells at metaphase due to the activation of the spindle checkpoint, caused by the loss of β-tubulin function. The product of the cold-sensitive allele *nda3*-KM311 resumes its ability to polymerize into microtubules within 6 minutes of return to the permissive temperature [43] and allows progression through chromosome segregation and mitotic exit.

**Figure 2:**
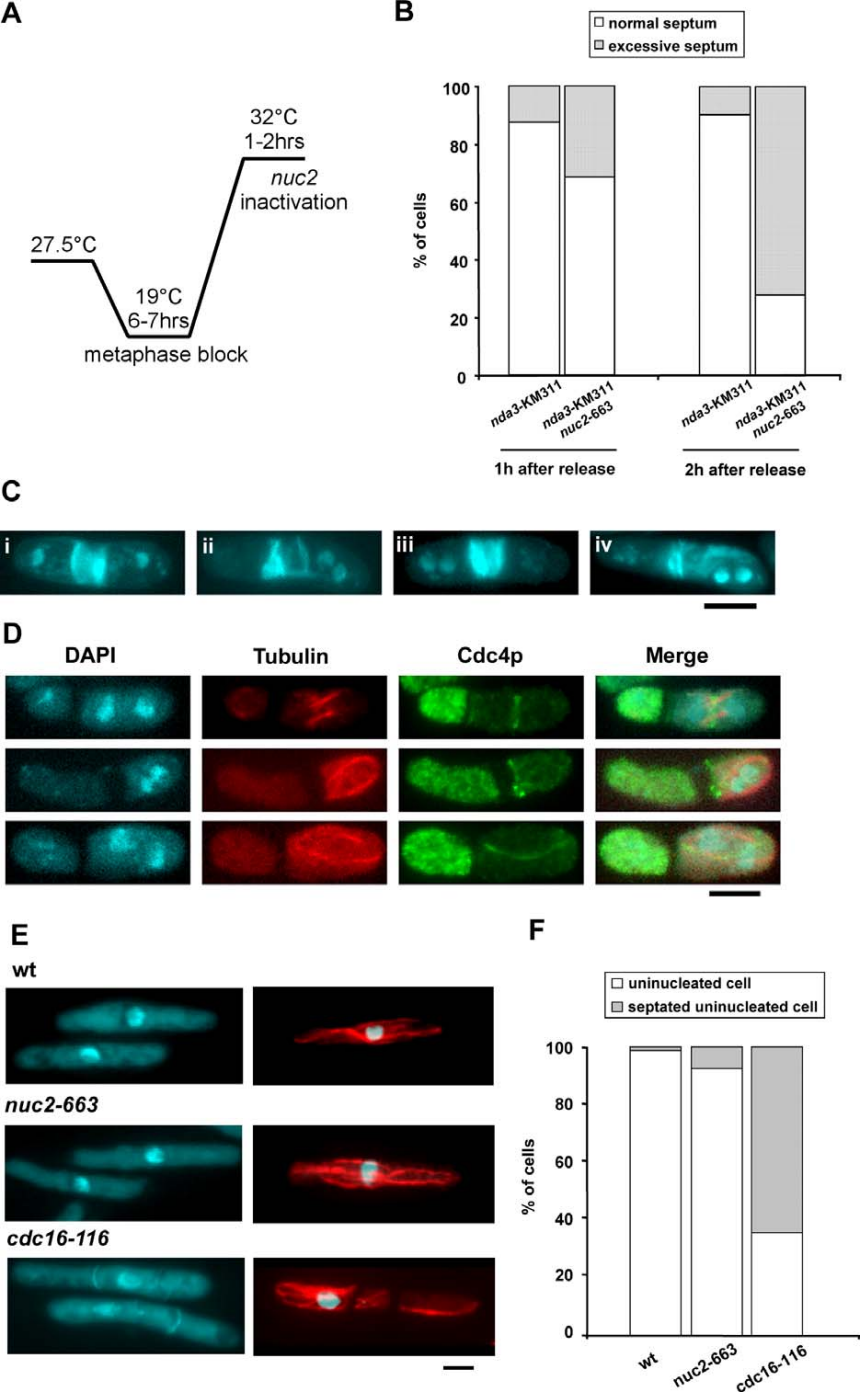
Ectopic Actomyosin Ring and Septum Formation in the *nuc2-*663 Mutant After Septation (A) The diagram schematically illustrates the experimental design. (B) Quantification of cells with normal or excessive septa after 1–2 h release from metaphase block. At least 200 cells were counted in each category. (C) Examples of septum patterns in *nuc2-*663 *nda3-*KM311 cells. Four examples of cells with excessive septum material are shown. i and ii, multiseptated cells; iii, excessive deposition of septum material at division site; iv, cell with ectopic misoriented septum. (D) Visualization of actomyosin rings in *nuc2*-663 *nda3*-KM311 cells by immunofluorescence microscopy. Top, middle, and bottom panels show cells with actomyosin rings that were formed straight, close to, or misoriented with respect to the previous division site, respectively. Microtubules were stained with TAT-1 antibody, and the actomyosin ring was stained with anti-Cdc4p antibody. (E) Septum assembly in S-phase–arrested cells. Wild-type, *nuc2-*663, and *cdc16-*116 cells arrested in S phase by hydroxyurea treatment were either formaldehyde fixed for DAPI and aniline blue staining or methanol fixed for immunostaining with TAT-1. (F) Quantification of septated cells versus non-septated cells upon hydroxyurea treatment. At least 300 cells were counted for each category. Scale bar, 5 μm.

The *nuc2*-663 *nda3*-KM311 and *nda3*-KM311 (as a control) cells were first cultured at 19 °C to inactivate Nda3p function. Under these conditions, at least 50% of cells arrested at metaphase due to the activation of the spindle assembly checkpoint. Subsequently, these cells were shifted to 32 °C to inactivate Nuc2p function and to reactivate Nda3p function (Figure 2A). Since reactivation of Nda3p function (based on the immediate assembly of the mitotic spindle and 40% of total cells formed an elongated anaphase spindle after 45 min at 32 °C) occurred more rapidly than the inactivation of Nuc2p, *nuc2-*663 *nda3-*KM311 (and the control *nda3-*KM311) underwent anaphase and septation. Interestingly, maintenance of *nuc2-*663 *nda3-*KM311 at 32 °C led to the accumulation of cells that either formed multiple septa, or deposited excessive septum material in the vicinity of the first septum (Figure 2B and 2C, cells i–iii). Cells with mis-oriented ectopic septa were also frequently observed (Figure 2C, cell iv). These effects were observed less frequently in the control *nda3-*KM311 cells (Figure 2B). In addition to division septa, additional actomyosin rings (as visualized with antibodies against the myosin light chain Cdc4p) were also detected in *nuc2-*663 *nda3-*KM311 cells (Figure 2D). These rings were either orthogonally placed or mis-oriented (Figure 2D, Cdc4p panel). The fact that cells with actomyosin rings contained interphase microtubule arrays (Figure 2D, tubulin panel), suggested that the assembly of additional actomyosin rings was not linked to re-entry into mitosis.

The formation of excessive septum after one round of cytokinesis raised the possibility that SIN activation in *nuc2-*663 mutant might be able to trigger cytokinesis in interphase cells as has been observed in *cdc16-*116 mutants [3,39]. To test whether this is the case, *nuc2-*663 mutant and *cdc16-*116 mutant were treated with hydroxyurea to block cells in S phase and shifted to restrictive temperature to heat inactivate Nuc2p and Cdc16p functions. As previously reported, heat inactivation of Cdc16p function led to formation of septated cells during interphase as indicated by the interphase microtubule organization and presence of septum in these cells (Figure 2E, *cdc16*-116 panel, and 2F). The *nuc2-*663 mutant at restrictive temperature, however, was similar to wild-type cells and did not assemble division septa (Figure 2E, *wt* and *nuc2*-663 panels, and 2F). The observation that interphase arrested *nuc2-*663 cells did not assemble division septa suggested that Nuc2p might play an important role in preventing additional rounds of cytokinetic events following septation.

Since Nuc2p is a subunit of the APC/C, it was possible that the entire APC/C might function to inhibit inappropriate cytokinesis. Alternatively, it was possible that Nuc2p regulated cytokinesis in a manner independent of other subunits of the APC/C. To distinguish between these possibilities, we assayed the ability of *cut9*-665 and *lid1*-6 (two essential components of the APC/C [44,45]) mutants to accumulate multiple septa. As before, we tested for the presence of multiple and excessive septa upon shift of synchronous *nda3*-KM311 *cut9*-665 and *nda3*-KM311 *lid1*-6 to conditions that inactivated the APC/C components. In these experiments, *nda3*-KM311 *cut9*-665 and *nda3*-KM311 *lid1*-6 behaved similar to *nda3*-KM311 cells and did not accumulate multiple and excessive septa (Figure S1). Furthermore, activation of APC/C by overexpression of Slp1p (Figure S1B) or Ste9p [46,47] did not lead to septation defects, indicating that APC/C activation per se does not lead to septation defects. Collectively, these experiments suggested that the inhibition of inappropriate cytokinesis by Nuc2p might not require the other subunits of the APC/C.

### Cells Overexpressing Nuc2p Assemble, but Do Not Maintain, Actomyosin Rings

**Figure 3:**
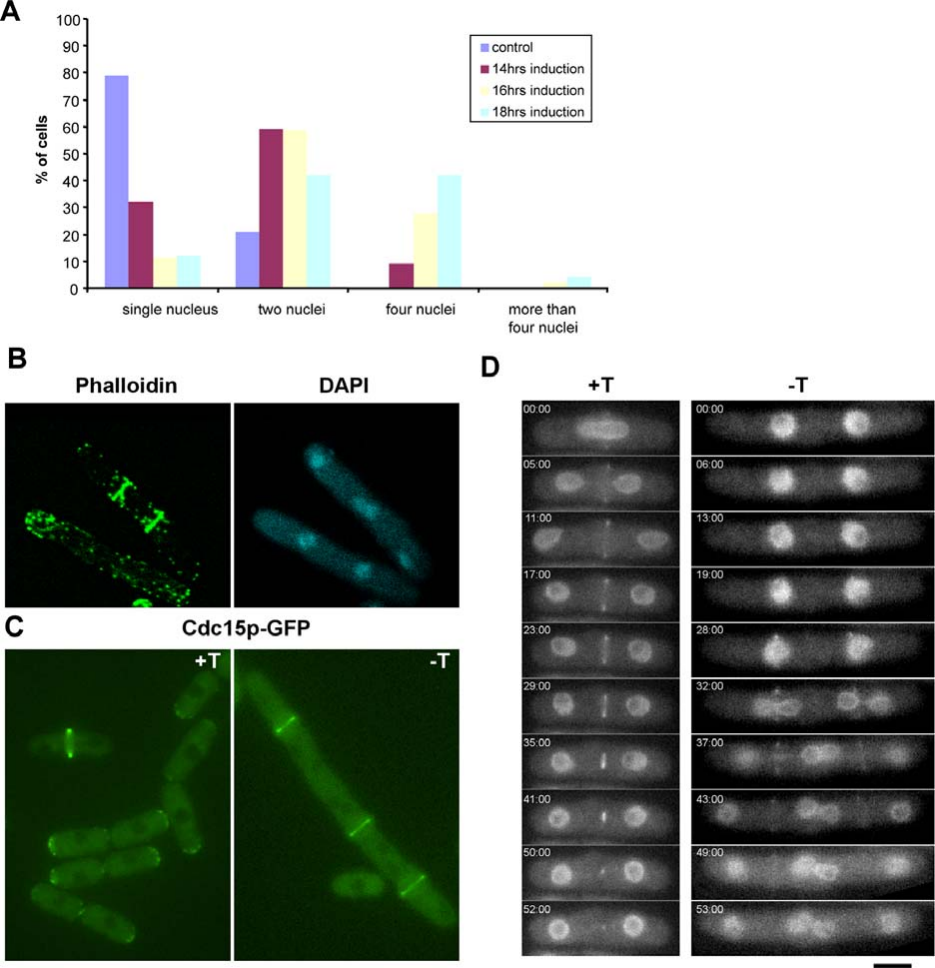
Actomyosin Rings Are Assembled, but Not Maintained, at the Division Site in Cells Overexpressing Nuc2p (A) Quantification of the number(s) of nuclei/cell and the frequency of their appearance upon overexpression of Nuc2p. Cells expressing Uch2p-GFP as a nuclear marker were used in the experiment. (B) Visualization of F-actin. Cells overexpressing Nuc2p were fixed and stained with phalloidin and DAPI to visualize the F-actin cytoskeleton and nuclei, respectively. (C) Visualization of Cdc15p. Cells carrying Cdc15p-GFP were induced to overexpress Nuc2p and visualized by fluorescence microscopy. +T/−T indicates medium supplemented with or without thiamine. (D) Time-lapse fluorescence microscopy to image the dynamics of actomyosin ring assembly and constriction. Cells were grown in medium with or without thiamine and imaged by time-lapse microscopy. The nucleus and actomyosin ring were marked by Uch2p-GFP and Rlc1p-GFP, respectively. Scale bar, 5 μm.

We have found that loss of Nuc2p function leads to persistent localization of SIN components, such as Cdc7p, Sid1p, and Sid2p, even after completion of septation. Previous studies have shown that overproduction of Nuc2p leads to defective cytokinesis, although the basis of this effect remained unknown [40]. To gain insights into the mechanism by which Nuc2p overproduction inhibits cytokinesis, a strain of yeast was generated in which the thiamine-repressible promoter, *nmt1*, was used to replace the endogenous *nuc2* promoter (hereafter referred to as *nmt1-nuc2*). The *nmt1-nuc2* strain was used in all overexpression experiments. The *nmt1-nuc2* strain resembled wild type cells in morphology upon growth in medium supplemented with thiamine. To analyze the overexpression phenotype more thoroughly, we generated an *nmt1-nuc2* strain that expresses the nuclear marker Uch2p-GFP (Ubiquitin C-terminal hydrolase fused to GFP). When this strain was grown in medium containing thiamine, cells were found to contain either a single nucleus or two nuclei (Figure 3A), depending on the cell cycle stage. Upon removal of thiamine from the medium (leading to overexpression of Nuc2p), a high proportion of cells accumulated two or more nuclei (Figure 3A). In particular, after 18 h of derepression of the *nmt1* promoter, more than 40% of the cells contained 4 or more nuclei, while another 40% of the cells contained two nuclei, most of which were of a post-mitotic configuration. We also attempted to overproduce Nuc2p in synchronous cultures, but these experiments did not lead to significant synchrony at the point of maximal induction of *nmt1* promoter, due to the long amount of time required to fully derepress the *nmt1* promoter (unpublished data).

It is known that cytokinesis in fission yeast requires the function of an actomyosin ring [16]. We considered the possibility that overexpression of Nuc2p affects the assembly of actin and/or myosin components at the cell division site. To address this question, we grew the *nmt1-nuc2* cells in medium lacking thiamine to overexpress Nuc2p and stained these cells with phalloidin. In cells overexpressing Nuc2p, F-actin assembled into ring structures in mitotic cells, as in control cells, suggesting that the recruitment and assembly of F-actin into the actomyosin ring was not affected (Figure 3B). In addition, we tested the effect of Nuc2p overexpression on the localization of the FCH domain protein, Cdc15p, which is essential for actomyosin ring maintenance and septum assembly [10,48,49]. As in the case of F-actin, cells were able to assemble Cdc15p rings upon Nuc2p overexpression (Figure 3C).

To observe the dynamics of ring assembly and constriction process, we performed time-lapse microscopy in *nmt1-nuc2* cells expressing Rlc1p-GFP (regulatory light chain of myosin fused to green fluorescent protein) and Uch2p-GFP as the markers for actomyosin ring and nucleus, respectively. Under conditions of Nuc2p overexpression, cells assembled Rlc1p rings overlying the position of the interphase nuclei (Figure 3D, −T panel). However, these rings were not stable and collapsed in late anaphase and failed to constrict, leading to failure of septation (Figure 3D, −T panel). In contrast, the Rlc1p rings in control cells constricted normally at the end of mitosis leading to the formation of a division septum (Figure 3D, +T panel). Collectively, these studies established that the presence of excess Nuc2p led to defects in maintenance of actomyosin rings in late anaphase, leading to failure of division septum assembly.

### Overexpression of Nuc2p Leads to Loss of Cdc7p and Sid1p from the SPB

**Figure 4:**
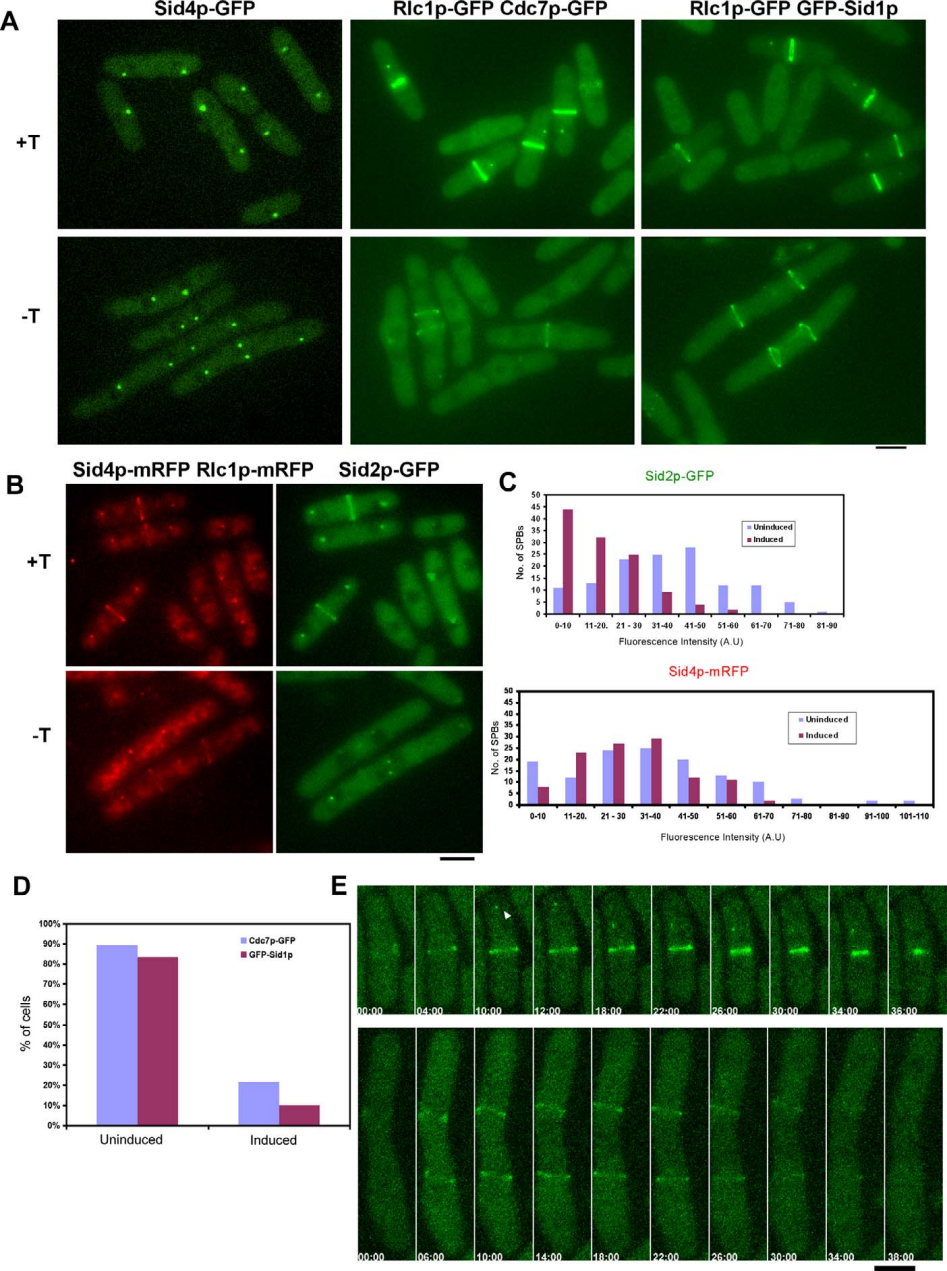
Overexpression of Nuc2p Inhibits SIN Signaling (A) *nmt1-nuc2* cells expressing Sid4p-GFP, Cdc7p-GFP, and GFP-Sid1p were visualized by fluorescence microscopy in the presence (+T, top panel) or absence (−T, bottom panel) of thiamine. The actomyosin ring (visualized with Rlc1p-GFP) was used as the marker for cells undergoing cytokinesis. (B) Fluorescence signal of Sid2p is reduced in cells overproducing Nuc2p. *nmt1-nuc2* cells expressing Sid2p-GFP were visualized using fluorescence microscopy upon growth on medium supplemented with or without thiamine. SPBs and actomyosin ring were marked by Sid4p-mRFP and Rlc1p-mRFP, respectively. (C) Quantification of the relative fluorescence intensities of Sid2p-GFP and Sid4p-mRFP in Nuc2p-uninduced and -induced cells. (D) Quantification of actomyosin ring containing cells with Cdc7p-GFP or GFP-Sid1p at the SPBs. At least 80 cells were counted for each category. (E) Cdc7p is not recruited to the SPBs in cells overproducing Nuc2p. Top panel, control cell grown on minimal medium supplemented with thiamine. Arrowhead indicates localization of Cdc7p-GFP at the SPB. Bottom panel, cell grown in medium without thiamine to overexpress Nuc2p. Rlc1p-GFP was used in these cells to label actomyosin ring. Cells were grown on agarose pad containing growth medium and were imaged by laser scanning microscopy. Scale bar, 5 μm.

Previous studies have shown that the mutants defective in the septation initiation network (SIN) assemble actomyosin rings, but these rings collapse late in anaphase, leading to a defect in division septum assembly [16,34]. Since we observed a phenotype similar to that seen in SIN mutants in cells overexpressing Nuc2p and a complementary phenotype in *nuc2-*663 cells, it was possible that overexpression of Nuc2p might affect SIN signaling. To determine whether this was the case, we examined the localization of SIN components Sid4p, Cdc11p, Spglp, Cdc7p, Sid1p, and Sid2p upon Nuc2p overexpression. Of these proteins, Sid4p, Cdc11p, Spg1p, and Sid2p are known to localize to the SPBs throughout the cell cycle, while Cdc7p and Sid1p localize during cytokinesis to one of the two SPBs (which has been shown to contain GTP-bound Spg1p) [18]. The localization of Sid4p, Cdc11p, and Spg1p to the SPBs was unaltered upon overexpression of Nuc2p (Figure 4A and our unpublished observation). Interestingly, the localization of Cdc7p and Sid1p was severely affected in mitotic cells overexpressing Nuc2p. Cdc7p was detected at the SPBs in 12/61 cells with actomyosin rings, while Sid1p was detected in 6/71 cells with rings (Figure 4A and 4D, −T and Induced panels), compared to uninduced cells in which at least 80% of cells with actomyosin rings contained Cdc7p-GFP and GFP-Sid1p at the SPBs (Figure 4D). Furthermore, in Nuc2p overproducing cells that did display Cdc7p and Sid1p at the SPBs, the signal of Cdc7p and Sid1p was significantly reduced compared to that observed in control cells (Figure 4A). Consistent with the loss of Cdc7p and Sid1p from the SPBs, the fluorescence signal of the most downstream kinase of the SIN, Sid2p, was dramatically reduced upon overexpression of Nuc2p (Figure 4B and quantification of Sid2p-GFP fluorescence in relation to Sid4p-mRFP is shown in Figure 4C).

Next, we determined if overexpression of Nuc2p affected the recruitment or the maintenance of SIN components at the SPBs. To this end, we performed time lapse microscopy to observe the localization of Cdc7p during cytokinesis. We found that Cdc7p was recruited to one of the SPBs during actomyosin ring constriction in cells grown in the presence of thiamine (Figure 4E, top panel; indicated by arrowhead). In contrast, in cells overexpressing Nuc2p, Cdc7p was not detected at the SPBs throughout the cell division cycle and the actomyosin ring disassembled at the end of mitosis (Figure 4E, bottom panel). Taken together, these studies suggested that overexpression of Nuc2p affects the recruitment of the SIN components, Cdc7p and Sid1p to SPB.

### Overexpression of Nuc2p Affects the Binding of Spg1p and Cdc7p, but Not the Steady-State Levels of Cdc7p, Sid1p, and Sid2p

**Figure 5:**
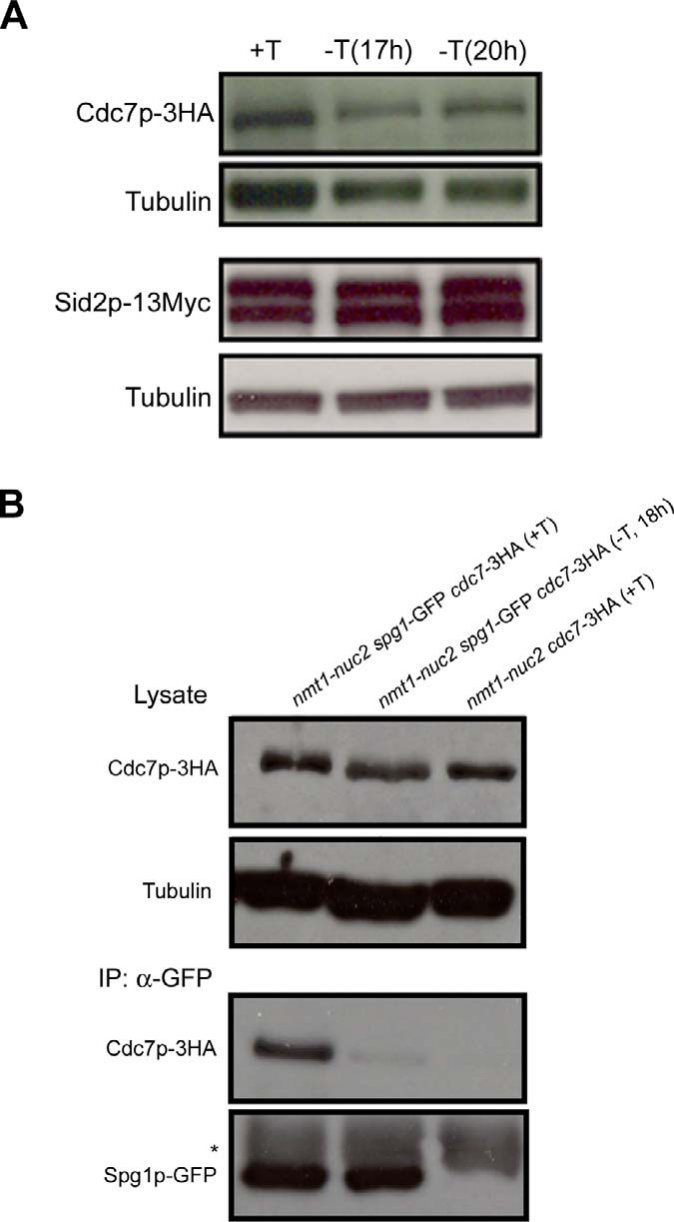
Overexpression of Nuc2p Does Not Affect the Steady-State Levels of Cdc7p-3HA and Sid2p-13Myc, but Disrupts the Binding of Spg1p-GFP and Cdc7p-3HA (A) Protein levels of Cdc7p-3HA and Sid2p-13Myc in *nmt1-nuc2* cells grown in medium with or without thiamine. Tubulin serves as the loading control. (B) Lysates from cells expressing Cdc7p-3HA and Spg1p-GFP in the presence or absence of Nuc2p overexpression were immunoprecipitated with GFP antibodies and immunoblotted with antibodies against the HA epitope. Lysates from a strain without Spg1p-GFP was included as the control. Tubulin serves as the loading control. Asterisk indicates heavy chain of IgG.

Since Nuc2p is a component of the APC/C, and participated in degradation of molecules regulating mitosis, we considered the possibility that overproduction of Nuc2p might lead to instability and degradation of one or more of the SIN components. We therefore tested the steady state levels of the SIN components Cdc7p, Sid1p, and Sid2p in cells overexpressing Nuc2p. We found that the steady state levels of Cdc7p, Sid1p, and Sid2p were not significantly altered upon overexpression of Nuc2p (Figure 5A; and unpublished observations on Sid1p). Thus, Nuc2p appears to affect the SPB localization of the SIN kinases but not their stability, consistent with our findings that other subunits of APC/C were not required for the inhibition of septation by Nuc2p.

It has been shown that the small GTPase Spg1p binds to Cdc7p and recruits this protein kinase to SPBs during mitosis. Cdc7p binds preferentially to GTP-bound Spg1p, which is thought to be the activated form of this GTPase [35,50]. The fact that the recruitment of Cdc7p to the SPBs was affected in Nuc2p overexpressing cells suggested that the binding between Spg1p and Cdc7p might be affected upon Nuc2p overproduction. To test if this was the case, we overproduced Nuc2p in cells expressing Spg1p-GFP and Cdc7p-3HA and performed co-immnunoprecipitation experiments to look for a physical interaction between Spg1p and Cdc7p. Control immunoprecipitation experiments were carried out from lysates prepared from cells expressing Cdc7p-3HA (but not Spg1p-GFP). Immunoblotting with HA antibodies showed that the level of Cdc7p was comparable in both strains used as well as under both conditions (+ or – thiamine) used (Figure 5B). Immunecomplexes generated with GFP antibodies contained Cdc7p when cells were grown in medium containing thiamine (Figure 5B, +T of IP panel). Interestingly, immunecomplexes generated with GFP-antibodies from cells overproducing Nuc2p contained very little or no Cdc7p-HA (Figure 5B, −T of IP panel). Immune complexes generated with GFP antibodies from cells expressing Cdc7p-HA, but not Spg1p-GFP, did not contain Cdc7p-HA, establishing the specificity of the immunoprecipitation procedure. These experiments established the binding between Cdc7p-3HA and Spg1p-GFP was interrupted in cells overproducing Nuc2p.

### Genetic Evidence that Nuc2p Affects Byr4p-Cdc16p Function

**Figure 6:**
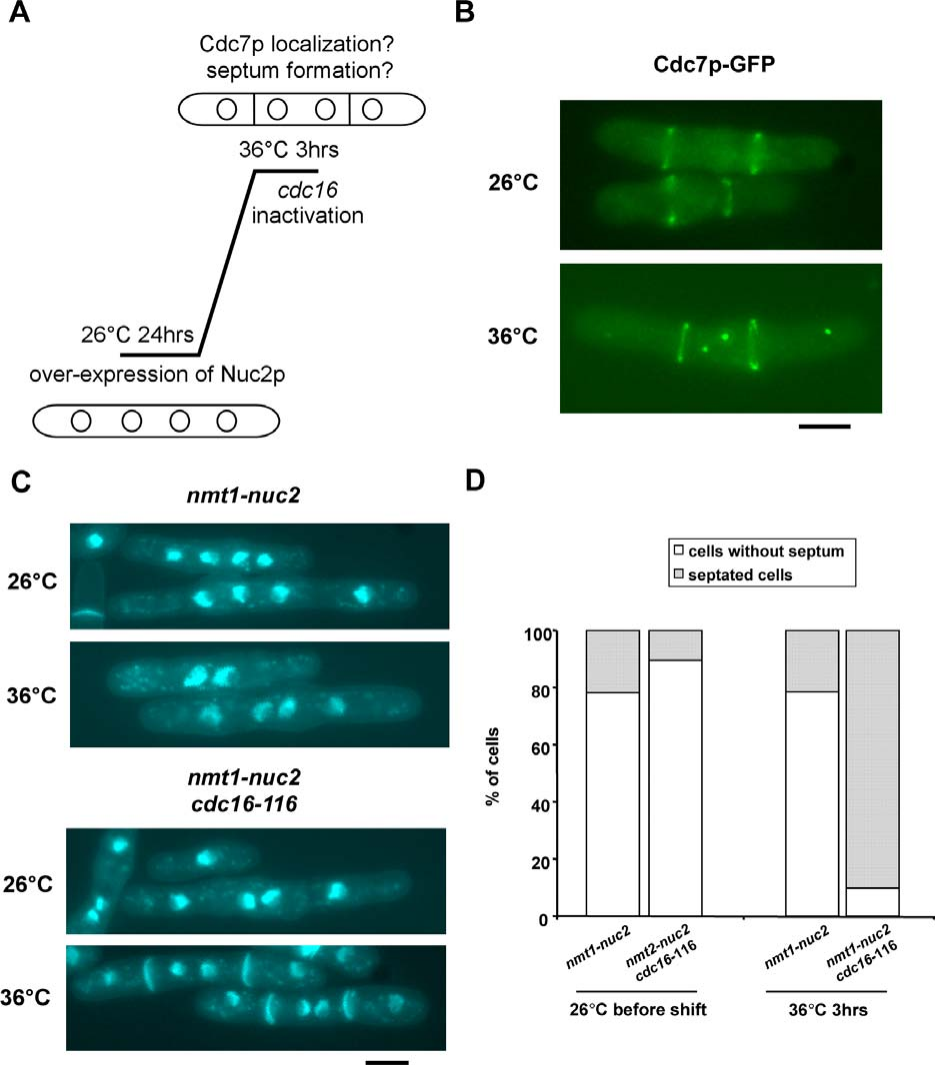
Inactivation of Cdc16p Function Promotes Localization of Cdc7p to SPBs and Allows Septation in Cells Overexpressing Nuc2p (A) Diagram schematically illustrates the experimental design. Nuc2p was induced for 24 h at 26 °C, and the culture was shifted to 36 °C to inactivate Cdc16p and examined after 3 h of incubation. (B) The localization of Cdc7p-GFP in *cdc16*-116 cells overexpressing Nuc2p was visualized by fluorescence microscopy at the permissive or restrictive temperatures. (C) Septum assembly is restored upon inactivation of Cdc16p in cells overexpressing Nuc2p. DAPI and aniline blue staining of formaldehyde fixed cells is shown. (D) Quantification of septated cells versus non-septated cells in *nmt1-nuc2* and *nmt1-nuc2 cdc16-*116. Note that only cells with four nuclei were counted. Scale bar, 5 μm.

We have shown that cells overproducing Nuc2p are able to localize Sid4p, Cdc11p, and Spg1p to the SPBs. In contrast, the localization of Cdc7p, Sid1p and Sid2p-Mob1p was significantly reduced/altered upon overproduction of Nuc2p. Cdc7p has been shown to preferentially bind to GTP-bound Spg1p [35], and we have shown that overproduction of Nuc2p leads to a reduction/failure of physical interaction between Spg1p and Cdc7p. Thus it was possible that overproduction of Nuc2p led either to the inactivation of putative guanine nucleotide exchange factors (GEF) for Spg1p or to the persistent activation of the two-component GAP (Byr4p-Cdc16p), leading to the maintenance of Spg1p in its inactive GDP bound form. Since proteins related to the budding yeast Lte1p (functions as a GEF for the Tem1p-GTPase, which is related to fission yeast Spg1p) [51], have not been identified in fission yeast, we considered the possibility that overproduction of Nuc2p might cause activation of Byr4p-Cdc16p. We tested this idea by overproduction of Nuc2p in *cdc16-*116 mutants and assayed the ability of these cells to localize Cdc7p to the SPB and to assemble division septa (Figure 6A). Inactivation of Cdc16p function in cells overexpressing Nuc2p resulted in the recruitment/maintenance of Cdc7p on SPBs (Figure 6B, bottom panel), whereas this was not detected in the presence of Cdc16p function (Figure 6B). More than 90% of the tetranucleate cells were able to undergo cytokinesis and septation (*n* = 190/203, Figure 6C and 6D). In contrast, less than 25% of tetranucleate cells in which Cdc16p was functional underwent cytokinesis and septation (*n* = 44/204, Figure 6C and 6D). Although other interpretations are possible, we favour the idea that overproduction of Nuc2p might lead to activation of Byr4p-Cdc16p, thereby to the inability to maintain SIN function and septation.

### Nuc2p Functions Independently of CHFR-Related Dma1p To Inhibit the SIN

**Figure 7:**
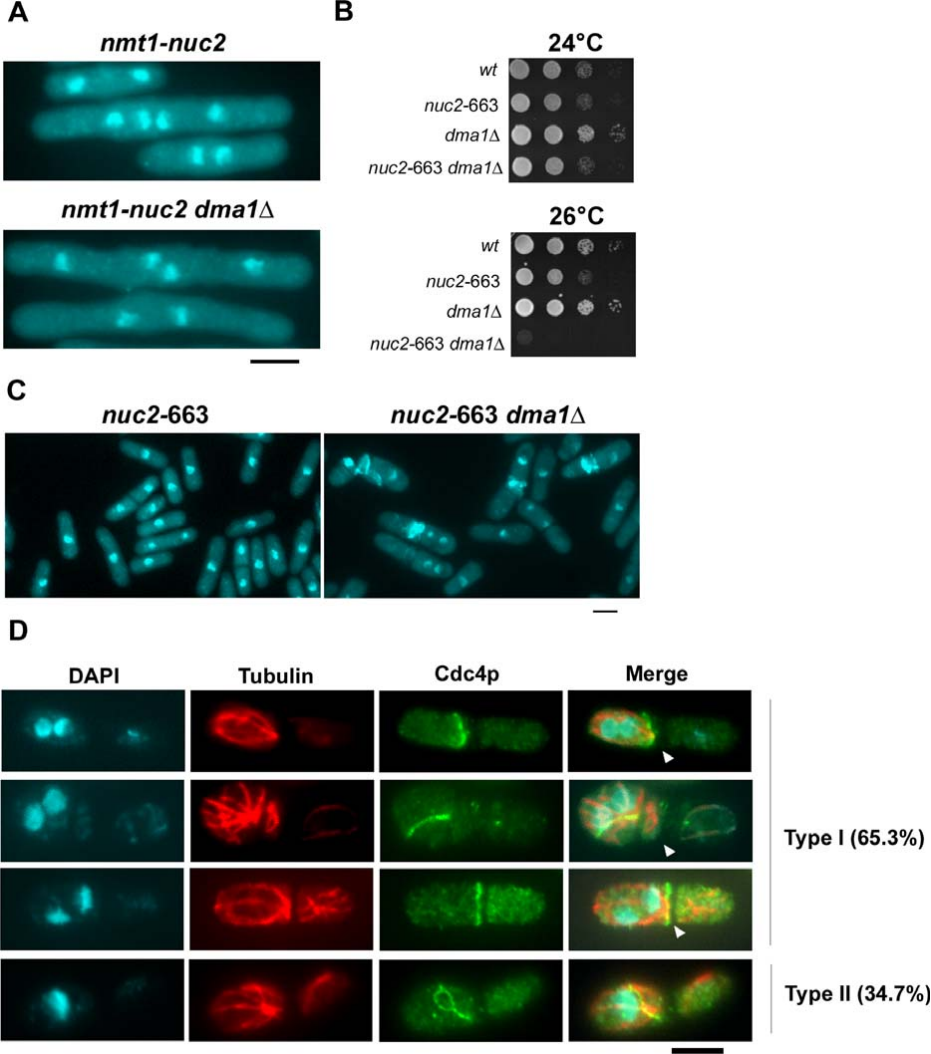
Nuc2p Acts Independently of Dma1p to Regulate Cytokinesis (A) Overexpression of Nuc2p in wild-type or *dma1*Δ mutant. (B) Wild-type, *nuc2-*663, *dma1*Δ, and *nuc2-*663 *dma1*Δ strains were diluted serially and spotted on YES agar and incubated at 24 °C or 26 °C. (C) Septum patterns in *nuc2-*663 and *nuc2-*663 *dma1*Δ mutants grown at 28 °C. Cells were fixed and stained with DAPI and aniline blue. (D) Visualization of actomyosin rings and microtubules in *nuc2*-663 *dma1*Δ cells by immunofluorescence microscopy. Microtubules were stained with TAT-1 antibody and the actomyosin ring was stained with antibodies against Cdc4p. About 150 cells were counted in each category. Arrowhead indicates septum assembled in previous cell division.

Previous studies have shown that overproduction of Dma1p, a fission yeast protein related to human CHFR, leads to inhibition of SIN function and defective cytokinesis [52,53]. Since overproduction of Nuc2p also led to defects in SIN signaling, we tested if the cytokinesis-inhibitory effect upon Nuc2p overproduction depended on Dma1p. To this end, Nuc2p was overproduced in cells lacking Dma1p. Cytokinesis defects were observed in control (*dma1* ^+^) cells as well as *dma1*Δ cells overproducing Nuc2p (Figure 7A). This experiment suggested that the SIN-inhibitory effect caused by overproduction of Nuc2p was independent of Dma1p. To firmly establish if this was the case, double mutants defective in *nuc2* and *dma1* were constructed. Interestingly, whereas *nuc2*-663 and *dma1*Δ cells were able to form colonies at 26 °C, the double mutants were unable to do so (Figure 7B). Staining of DNA and septum material revealed that the *nuc2*-663 *dma1*Δ, but not *nuc2*-663, cells displayed an aberrantly septated phenotype, similar to that observed in *nuc2*-663 cells at higher temperatures (Figure 7C). Immunostaining of *nuc2*-663 *dma1*Δ cells with antibodies against Cdc4p and tubulin revealed that 65.3% of septated cells contained segregated DNA, interphase microtubules and actomyosin cables/rings (Figure 7D, type I). Furthermore, 34.7% of the septated cells contained unsegregated DNA, interphase microtubules, and actomyosin rings/cables (Figure 7D, type II). Since the multi-septate phenotype is largely detected in cells in which chromosome segregation is not affected, we conclude that the effect of septation in the *nuc2*-663 *dma1*Δ double mutants is not purely due to synthetic effects on APC/C function. Collectively, these studies established that Nuc2p and Dma1p inhibited the SIN by different mechanisms.

## Discussion

Previous studies have characterized the role of Nuc2p, a TPR-containing subunit of APC/C, in the regulation of progression through events of mitosis [44,54,55]. Previous studies have also proposed that Nuc2p might function as an inhibitor of septation, although the mechanism of this inhibition was not investigated [40]. In the current study we show that Nuc2p prevents septum assembly by inhibiting the septation initiation network (SIN).

Cells defective for *nuc2* display a “cut” phenotype, characterized by septum assembly in the absence of proper segregation of chromosomes [41]. Interestingly, we have found that prolonged incubation of *nuc2*-663 mutants at the restrictive temperature leads to the accumulation of cells with multiple septa. The specific effects of Nuc2p on septation are particularly clear in *nuc2*-663 mutants shifted to the restrictive temperature after execution of Nuc2p function in mitosis. The multiseptate phenotype of *nuc2*-663 is reminiscent of that displayed by cells in which the SIN is constitutively activated, such as in cells defective in Cdc16p, which together with Byr4p functions as a GTPase activating protein for the Spg1p-GTPase [28,29]. As in the case of *cdc16*-116 cells, the SIN components Cdc7p and Sid1p are retained at the SPBs even after septum assembly in *nuc2*-663 cells. In addition, Sid2p, considered to be the most downstream element of the SIN, is retained at the cell cortex after septum assembly in *cdc16*-116 and *nuc2*-663 mutants. Interestingly, overproduction of Nuc2p has been shown to cause defects in cytokinesis ([40] and this study). Furthermore, overproduction of Nuc2p leads to defects in actomyosin ring maintenance and localization of Cdc7p, Sid1p, and Sid2p to the SPB, suggestive of defective SIN signaling. Thus, while *nuc2* mutants phenocopy *cdc16* mutants (in which SIN is constitutively active), overproduction of Nuc2p leads to a phenotype indistinguishable from that displayed by SIN-defective mutants. These results imply that Nuc2p might be a bona fide inhibitor of the SIN. Interestingly, whereas activation of SIN, by loss of Cdc16p function, promotes events of cytokinesis (actomyosin ring and division septum assembly) in premitotic interphase arrested cells, the loss of Nuc2p function in premitotic interphase does not lead to actomyosin ring and septum assembly. Thus, although Nuc2p appears to be an inhibitor of the SIN, Nuc2p might inhibit SIN specifically after cell division.

How does Nuc2p inhibit the SIN? Cells overexpressing Nuc2p phenocopy mutants defective in SIN function, which is a Spg1p GTPase driven signaling cascade. Interestingly, the localization of the upstream components of SIN, Cdc11p, Sid4p and Spg1p are not altered upon overproduction of Nuc2p. The increased level of Nuc2p, however, specifically affects the localization of downstream kinases: Cdc7p, Sid1p and Sid2p. Since Spg1p, but not its effectors Cdc7p and Sid1p, remains at the SPB, after Nuc2p overexpression, it can be speculated that the Spg1p in cells overexpressing Nuc2p is GDP-bound. Consistent with this, we have found that the physical interaction between Cdc7p and Spg1p is dramatically reduced in cells overproducing Nuc2p. Two possibilities can be envisaged for the mechanisms that maintain Spg1p in GDP-bound form. First, it is possible that overexpression of Nuc2p inhibits a putative guanine nucleotide exchange factor (GEF) for Spg1p, thereby preventing the loading of GTP onto Spg1p and the activation of SIN signaling. The second possibility is that Nuc2p promotes the activation of the two-component GTPase activating protein (GAP), Cdc16p-Byr4p, leading to the conversion of GTP-Spg1p into GDP-Spg1p. We do not favour the first possibility since no guanine nucleotide exchange factor for Spg1p has been identified to date. Our genetic analysis (restoration of Cdc7p localization and septum assembly in *cdc16*-116 cells overproducing Nuc2p) points to the second possibility that Nuc2p might function as an activator of Cdc16p-Byr4p. However, it also remains possible that Nuc2p might prevent the conversion of GDP-Spg1p to GTP-Spg1p by acting as a GDP dissociation inhibitor (GDI). Future studies should test these possibilities.

**Figure 8:**
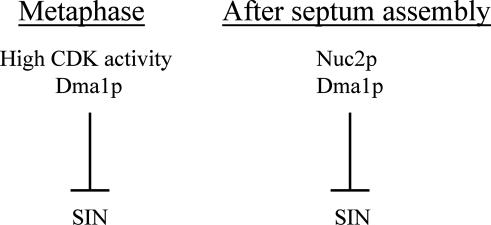
Model Summarizing the Temporal Regulation of Cytokinesis in S. pombe

What is the physiological function of the inhibition of SIN by Nuc2p? Both cell division and cell growth utilize actin cytoskeleton and its modulators. Persistent SIN signaling might sequester actin cytoskeleton at the division site and thereby block actin remodeling at the growth sites. Thus, prevention of cytokinesis after cell division might ensure proper growth polarity establishment. Several mechanisms have been uncovered in fission yeast that prevent cytokinesis by inhibiting SIN [4]. During metaphase, the human CHFR protein homolog in fission yeast, Dma1p, inhibits SIN signaling to prevent precocious cytokinesis [52,53]. Prior to mitotic exit (in metaphase and anaphase A), the inhibition of SIN function by high CDK activity ensures the coordination of mitosis and cytokinesis [22]. Our studies show that Nuc2p inhibits SIN function after completion of cytokinesis. Genetic analysis suggests that Nuc2p and Dma1p, both inhibitors of SIN, might function independently of each other. Our study also points to an additional function for Dma1p after cell division in the prevention of SIN activation. Thus, it appears that high CDK activity and Dma1p inhibit SIN during metaphase, while Nuc2p and Dma1p are required to prevent SIN activation and inappropriate cytokinesis after completion of cell division (Figure 8).

The current study shows that Nuc2p inhibits SIN signaling. However, it remains unclear whether Nuc2p acts independently or requires a specific set of APC/C components in order to inhibit septation. We have not been able to detect a multiseptate phenotype upon inactivation of function of two additional subunits of the APC/C, namely Cut9p and Lid1p, after passage through anaphase. These observations might suggest that Nuc2p, rather than the entire APC/C, is required for the inhibition of SIN following cell division. In addition, hyperactivation of APC/C by overexpression of Slp1p (this study) and Ste9p [46,47] does not lead to septation defects, suggesting that activation of APC/C does not lead directly to abnormal septation. In contrast, we have found that overproduction of Cut23p gives weak cytokinesis defects ([56] and our unpublished observation), suggesting a role for the entire APC/C in prevention of inappropriate cytokinesis. Collectively, our present studies lean toward a specific role for Nuc2p (rather than the entire APC/C) in the inhibition of cytokinesis. However, additional studies with stronger alleles of other APC/C components, which inactivate faster than the currently available alleles, might be required to firmly conclude if Nuc2p inhibition of the SIN requires the entire APC/C.

## Materials and Methods

### S. pombe strains, media, and reagents.

S. pombe strains used in this study are listed in Table 1. YES medium or Minimal medium with appropriate supplements were used to culture fission yeast cells. Strains were constructed by either random spore germination method or by tetrad dissection. The *nmt1-nuc2* strain was created by transforming a DNA fragment generated by Polymerase Chain Reaction (PCR) using the forward primer: 5′CAATAACAACCACCTGTTTGTACCCACATGTTTTTGTTGACATTAACTCCCATCGTTTCCAAAACTTTAATAGATTTGTCGAATTCGAGCTCGTTTAAAC3′ and the reverse primer: 5′CGTTCTGAATAAAAAATTGAATTATCATAATTCTGATTATCAATGCAATACCATATTAAACATTTCAATCGATCTGTCATCATGATTTAACAAAGCGACTATA3′. The positive clone was selected using Geneticin (Sigma) and confirmed by PCR. To overexpress Nuc2p in the *nmt1-nuc2* strain, cells were first grown in minimal medium containing 15 μM thiamine. The culture was then washed three times and re-inoculated into medium without thiamine, to induce Nuc2p expression.

**Table 1:**
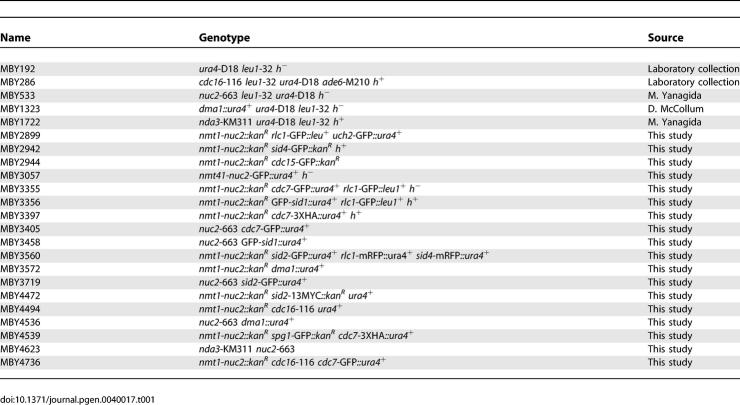
S. pombe Strains Used in This Study

To arrest cells in S phase, cells were treated with 12 mM hydroxyurea (HU; Sigma) for 6 h prior to any experimental manipulation. To synchronize *nuc2-*663 mutant at metaphase, *nda3-*KM311 (control) and *nuc2-*663 *nda3-*KM311 mutants were first grown at 27.5 °C in YES medium supplemented with 1.2 M sorbitol. Cells were washed three times with YES before shifting to 19 °C in YES medium to achieve metaphase-arrest.

### Gene tagging.

To fuse Rlc1p and Sid4p with monomeric red fluorescence protein (mRFP), DNA fragments containing either the *rlc1* or *sid4* were first amplified by PCR and were then cloned into pJK210-mRFP. The products were linearized using restriction enzymes and transformed into wild type S. pombe by the lithium acetate method [57]. DNA fragments containing C-terminal sequences of *rlc1* and *sid4* were amplified by PCR with primer pairs MOH461 5′GAGAGCTGGTACCTGAATGTTCTCTTCGAAGGAA3′ and MOH462 5′GAGAGTGCCCGGGATTGCTATCTTTTGACCC3′; MOH2460 5′CGGGGTACCTAAGGAGATGAATGCCACAATACAATC3′ and MOH2461 5′TCCCCCGGGCAAACTACGTTTTTTAAGCTCCC3′, respectively, for cloning.

### Immunoprecipitation and Western blotting.

Immunoprecipitation and Western blotting was performed as described [58]. Briefly, cell extracts were prepared by glass bead disruption and solubilised in buffer containing 1% Triton X-100, 150 mM NaCl, 2 mM EDTA, 6 mM Na_2_HPO_4_, 4 mM NaH_2_PO_4_, and complete protease inhibitors (Roche Diagnostics). Cell extracts were then clarified by centrifugation at 14,000 rpm for 10 min at 4 °C. To immunoprecipitate protein complex, 500 μl of soluble protein was incubated with 5 μl of -GFP antibodies for 1–2 h at 4 °C. Protein A-Sepharose beads (100 μl, Amersham Biosciences) were then added to the antigen-antibody immunecomplex and incubated for 45 min at 4‘°C. After six washes with buffer containing 1% Triton X-100, the beads were resuspended in SDS-PAGE loading buffer and heated at 95 °C for 5 min. The Protein A-Sepharose beads were spun down at 14,000 rpm for 5 min and the supernatants were subjected to SDS-PAGE. To detect GFP, Myc, or HA-tagged proteins, antibodies recognizing GFP, Myc (Sigma), or HA (Sigma) were used to probe the PVDF membranes containing separated proteins.

### Microscopy.

To visualize the F-actin cytoskeleton, cells were fixed with 7% formaldehyde and stained with Alexa Fluor-488 phalloidin (Molecular Probes). Septum/cell wall and DNA were stained with aniline blue (Sigma) and 4′, 6-diamidino-2-phenylindole (DAPI), respectively. For immunofluorescence studies, cells were fixed either with formaldehyde or with methanol. Cells were then processed as described [59]. Antibodies against Cdc4p and β-tubulin were used to stain the actomyosin ring and microtubules, respectively. Images were captured using an Olympus IX71 microscope equipped with a Photometrics CoolSNAP ES camera. All images were processed with MetaMorph 6.1. For confocal imaging, Zeiss LSM 510 confocal microscope equipped with a 63×/1.4NA PlanApo objective lens was used.

## Supporting Information

Figure S1 Analysis of Septation in Other APC/C Mutants, *cut9*-665 and *lid1*-6, and Overexpression of Slp1p (A) Cells were first synchronized at metaphase using cold sensitive allele of β-tubulin, *nda3*-KM311. The cultures were shifted to 36 °C to release cells from the metaphase block and to inactivate Cut9p and Lid1p functions. Cells were then collected after 1 to 2 h release from the metaphase block and scored for septation phenotype (normal versus excessive septum) by staining the cells with DAPI and aniline blue.
(B) The APC/C activator Slp1p was overexpressed from *nmt1* promoter in wild-type cells. Shown are DAPI stained images of cells grown in the presence (repressing) and absence (inducing) of thiamine. Scale bar, 5 μm.
(1.7 MB TIF)

Table S1 S. pombe Strains Used in This Study (26 KB DOC)

## Abbreviations

APC/C: anaphase-promoting complex/cyclosome
CHFR: checkpoint with FHA and ring finger
SIN: septation initiation network
SPB: spindle pole body
TPR: tetratricopeptide repeat
