# Supplementary figures and images for: Nuc2p, a Subunit of the Anaphase-Promoting Complex, Inhibits Septation Initiation Network Following Cytokinesis in Fission Yeast
Source: PLoS Genet. 2008 Jan 25;4(1):e17. doi: 10.1371/journal.pgen.0040017 (PMC2213707; doi:10.1371/journal.pgen.0040017)

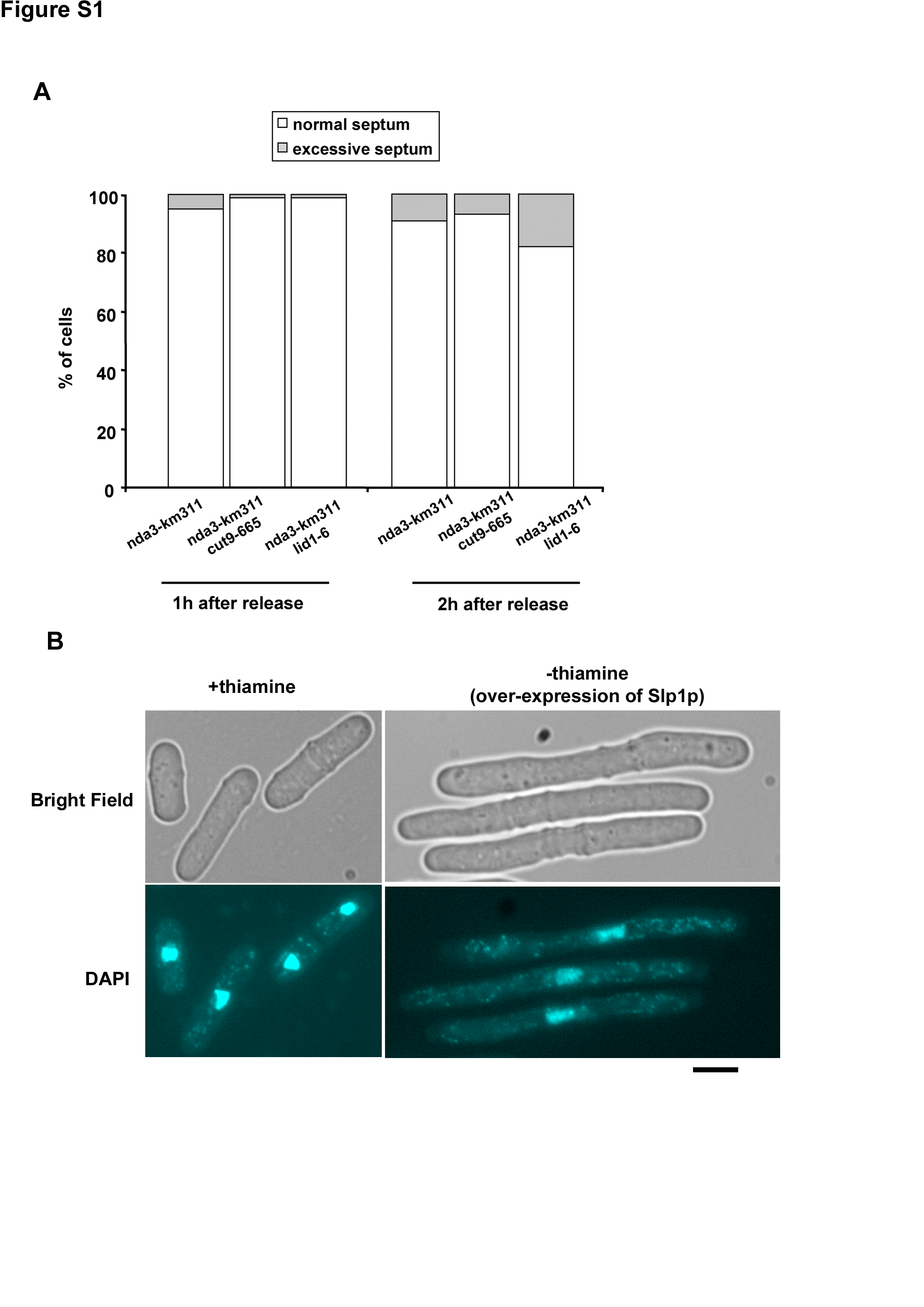

Supplement: Figure S1 — (A) Cells were first synchronized at metaphase using cold sensitive allele of β-tubulin, nda3-KM311. The cultures were shifted to 36 °C to release cells from the metaphase block and to inactivate Cut9p and Lid1p functions. Cells were then collected after 1 to 2 h release from the metaphase block and scored for septation phenotype (normal versus excessive septum) by staining the cells with DAPI and aniline blue. (B) The APC/C activator Slp1p was overexpressed from nmt1 promoter in wild-type cells. Shown are DAPI stained images of cells grown in the presence (repressing) and absence (inducing) of thiamine. Scale bar, 5 μm. (1.7 MB TIF) [file pgen.0040017.sg001.tif]
