# Supplementary material for: Nuc2p, a Subunit of the Anaphase-Promoting Complex, Inhibits Septation Initiation Network Following Cytokinesis in Fission Yeast
Source: PLoS Genet. 2008 Jan 25;4(1):e17. doi: 10.1371/journal.pgen.0040017 (PMC2213707; doi:10.1371/journal.pgen.0040017)
Supplement: Table S1 — (26 KB DOC) [file pgen.0040017.st001.doc]

**Table S1: Schizosaccharomyces pombe strains used in this study**

| Name | Genotype | Source |
| --- | --- | --- |
| MBY1722 | *nda3-*KM311 *ura4-*D18 *leu1-*32 *h^+^* | Yanagida M. |
| MBY4927 | *nda3-*KM311 *cut9*-665 | This study |
| MBY4929 | *nda3-*KM311 *lid1-*6 | This study |
| MBY5043 | *pREP1-slp1*(pCDL1270) in MBY192 | This study |
